# Primary and Secondary Yield Losses Caused by Pests and Diseases: Assessment and Modeling in Coffee

**DOI:** 10.1371/journal.pone.0169133

**Published:** 2017-01-03

**Authors:** Rolando Cerda, Jacques Avelino, Christian Gary, Philippe Tixier, Esther Lechevallier, Clémentine Allinne

**Affiliations:** 1 CIRAD, UMR System, 2 place Pierre Viala, Montpellier, France; 2 CATIE, Program of Sustainable Agriculture and Agroforestry, Turrialba, Costa Rica; 3 CIRAD, UR Bioagresseurs, TA A-106—Avenue Agropolis, Montpellier, France; 4 IICA, AP 55, Coronado, San José, Costa Rica; 5 INRA, UMR System, 2 place Pierre Viala, Montpellier, France; 6 CIRAD, UPR GECO, TA B-26 / PS4—Boulevard de la Lironde—Montpellier, France; 7 ENSAT, Avenue de l'Agrobiopole, Auzeville-Tolosane, France; US Department of Agriculture, UNITED STATES

## Abstract

The assessment of crop yield losses is needed for the improvement of production systems that contribute to the incomes of rural families and food security worldwide. However, efforts to quantify yield losses and identify their causes are still limited, especially for perennial crops. Our objectives were to quantify primary yield losses (incurred in the current year of production) and secondary yield losses (resulting from negative impacts of the previous year) of coffee due to pests and diseases, and to identify the most important predictors of coffee yields and yield losses. We established an experimental coffee parcel with full-sun exposure that consisted of six treatments, which were defined as different sequences of pesticide applications. The trial lasted three years (2013–2015) and yield components, dead productive branches, and foliar pests and diseases were assessed as predictors of yield. First, we calculated yield losses by comparing actual yields of specific treatments with the estimated attainable yield obtained in plots which always had chemical protection. Second, we used structural equation modeling to identify the most important predictors. Results showed that pests and diseases led to high primary yield losses (26%) and even higher secondary yield losses (38%). We identified the fruiting nodes and the dead productive branches as the most important and useful predictors of yields and yield losses. These predictors could be added in existing mechanistic models of coffee, or can be used to develop new linear mixed models to estimate yield losses. Estimated yield losses can then be related to production factors to identify corrective actions that farmers can implement to reduce losses. The experimental and modeling approaches of this study could also be applied in other perennial crops to assess yield losses.

## Introduction

Crop losses due to pests and diseases are a major threat to incomes of rural families and to food security worldwide [1, 2]. Quantitative information on crop losses and a better understanding of their drivers have been mentioned as essential to (i) evaluating the efficacy of crop protection practices [3], (ii) assessing systems sustainability [4], (iii) making better decisions for integrated pest management [5], and (iv) evaluating the effectiveness of pest and disease regulation as an ecosystem service [6, 7].

For some authors, crop loss is the reduction of the crop yield, defined both in terms of quantity and quality, that can occur in the field (pre-harvest) or in the storage (post-harvest) due to biotic or abiotic factors [3, 8]. For others, crop loss also includes the decrease in the value and financial returns of the crop [9]. Furthermore, crop losses comprise primary and secondary losses. Primary crop losses are those caused in the specific year when pest and disease injuries occur; secondary crop losses are those resulting from negative impacts of pests and diseases of the previous year [10]. In annual crops, the inoculum accumulation of pathogens in soil or in seeds and tubers remaining from the previous year, can cause secondary losses. These losses however can be avoided by implementing crop rotations or chemical treatments. In perennial crops, premature defoliation or the death of stems and branches caused by leaf injuries lead to loss of vigor and decreased production (secondary losses) in subsequent years. In this case, such secondary losses cannot be avoided since they come from already damaged plants [10].

A first step to crop-loss assessment is the quantification of yield losses, defined as the difference between attainable yield and actual yield [9]. The attainable yield is the site-specific yield achieved under the geographic and ecological conditions (radiation, temperature, water, and soil nutrients) of the location, with the best available production techniques and without the influences of any yield-reducing factors such as pests and diseases [8, 11]. The actual yield is also a site-specific yield, achieved with the available practices at farm level and resulting from the influence of yield-reducing factors such as pests and diseases [9, 11].

Despite the importance of information on crop losses, the main reviews on the topic agree that efforts to quantify yield losses and analyze their causes have been scant [1, 3, 8, 12]. Estimated yield losses for major food and cash crops (rice, wheat, barley, maize, potatoes, soybeans, cotton, and coffee) at country and regional levels have been mainly based on information from literature and trials conducted by chemical companies [3, 11]. However, in addition to being limited, yield-loss assessments normally do not consider secondary yield losses, which means that the real situation is not addressed. This is an issue for all crops, and even more so for perennial crops such as coffee, for which we assume that secondary losses may have far-reaching consequences.

Quantification of yield losses in perennial crops is particularly complex because of (i) the typical biennial pattern of production in these crops, characterized by a repetitive cycle of high production one year and low production the following year [13, 14], and (ii) the sustained presence of pests and diseases along years, which is an issue specially under tropical climates, which have no marked cold season to disrupt the life cycles of pests and diseases, and where susceptible organs of plants, particularly leaves, are almost always available.

The ongoing coffee crisis in Latin America and the Caribbean highlights the need for reliable assessments of coffee yield loss for governments, banks, and development agencies in order to implement appropriate economic responses [2]. This crisis has been triggered by a severe outbreak of coffee leaf rust (*Hemileia vastarix* Berkeley and Broome) combined with the inefficient management of coffee plantations, which have led to a decrease in production estimated between 30% and 50% in some countries, negatively affecting the incomes and food security of families involved directly or indirectly with coffee activities [2, 15, 16]. The quantification or reliable estimation of yield losses is a major challenge and requires trustworthy methods [1, 4].

Our research was a first attempt aimed at (i) quantifying primary and secondary coffee yield losses through field experimentation, taking into account a three-year dataset on foliar pests and diseases of coffee, and (ii) identifying the most important predictors of coffee yields and yield losses, through structural equation modeling.

## Materials and Methods

### Conceptual model for coffee yield losses

**Fig 1:**
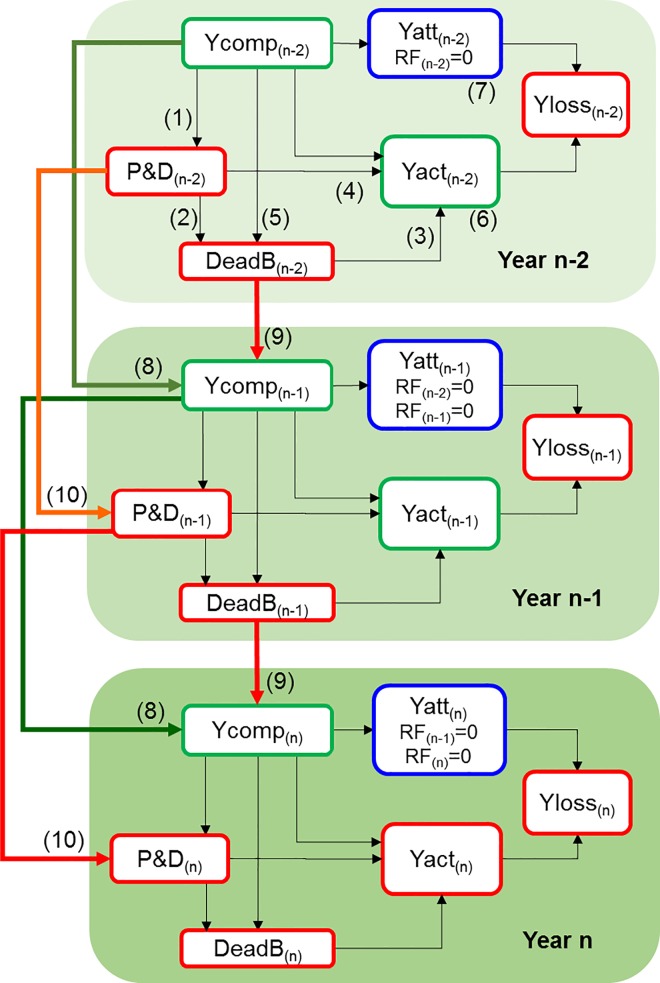
Conceptual model for the assessment of coffee yield losses caused by pests and diseases. Ycomp: yield components (productive stems and branches, fruiting nodes and fruits); P&D: pests and diseases; DeadB: dead productive branches; RF: reducing factors (P&D, DeadB); Yatt: attainable yield; Yact; actual yield; Yloss: yield loss; n: a given year. Within each year: (1) Yield components can affect pests and diseases, since it is known that high fruit loads can make the plant more susceptible to pathogens. (2) Pests and diseases can cause defoliation and thus contribute to the death of branches. (3) In turn, this will cause the drying and death of the fruits that were growing on them. (4) Pests and diseases can also reduce the photosynthetic capacity of the branch without causing its death but negatively affecting the development of fruits. (5) Yield components also influence the death of branches, because high fruit loads could cause the exhaustion of plant tissues, especially when the plant does not have enough nutrients to sustain growth and production. (6) The actual yield, then, as an output of the system, depends on yield components, pests and diseases, and dead productive branches. (7) The attainable yield would be the output of the system if there were no influences of pests and diseases (P&D = 0) nor dead branches (DeabB = 0), i.e., reducing factors = 0. Yield loss for a specific year (primary yield loss) is the difference between this attainable yield and the actual yield. Across years: (8) Yield components depend on the yield components of the previous year. A year with yield components achieving high values will be followed by a year with low values, and vice versa (biennial behavior of coffee production). (9) Yield components of the current year also depend on the number of dead branches of the previous year, as those branches will no longer be able to bear fruits. (10) Pest and disease abundance of the previous year will influence their abundance in the current year through its effect on primary inoculum. Secondary yield loss is the difference between the attainable yield (i.e., the yield with no yield-reducing factors in the previous and current years) and the actual yield obtained with reducing factors >0 in the previous year and reducing factors = 0 in the current year.

In order to guide the understanding of the experimental design and the statistical modeling, we developed a conceptual model for the assessment of coffee yield losses caused by pests and diseases. The model was built based on the scientific literature and the expert knowledge of the authors of this research. The model reflects effects over several years to assess secondary losses and take into account the already mentioned biennial behavior of coffee production [13, 17]. The model includes yield components, pests and diseases, and dead productive branches as the main yield drivers; attainable and actual yields are the outputs for the estimation of yield losses. Coffee yield is determined by the shoot growth of the previous year since fruits appear on the one-year-old wood [13]; pests and diseases can affect that shoot growth or cause the death of branches, compromising the yields of current and future years [2]. Here we consider that dead productive branches are those branches which had fruits but died mainly due to the impacts of pests and diseases (e.g. defoliation and dieback). Yield loss is derived from the difference between attainable and actual yield [9]. According to the effects of pest and disease injuries and dead branches, different actual yields, and therefore, different types of yield losses (primary and secondary) can be seen each year (Fig 1).

### Location and experimental design

We established an experimental coffee parcel with full-sun exposure on flat terrain at 648 masl (meters above sea level) on the farm of the Tropical Agricultural Research and Higher Education Center (CATIE) in Turrialba, Costa Rica. Turrialba is considered a rainy area, with slightly dry periods from March to April and rainiest periods in June and July. In the past 10 years, the mean annual rainfall was 2781 mm and the mean annual temperature was 22.2°C, with small variations among months.

**Fig 2:**
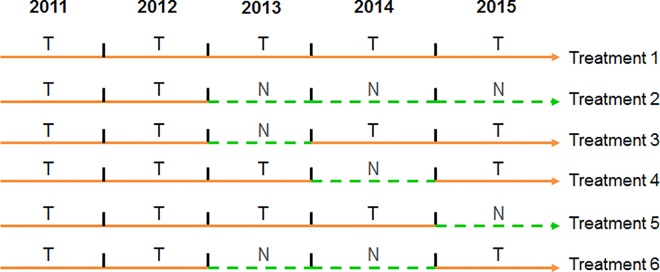
Scheme of the treatments applied in the coffee experimental parcel T: Treated with pesticides. Each year T consisted in: Fungicides: three applications of Opera^®^ (13.3% Pyraclostrobin and 5% Epoxiconazole) with doses = 2 ml lt^-1^; one of Soprano 25SC^®^ (12.5% Carbendazim and 12.5% Epoxiconazole) with doses: 1.25 ml lt^-1^; **Insecticides** (only in 2103)**:** three applications of Sumithion 50EC^®^ (50% Fenithrotion) with doses = 4 ml lt^-1^; one of Solver 48EC^®^ (48% Chlorpyrifos) with doses = 2.5 ml lt^-1^ **N: No pest and disease control**

The experimental parcel was planted in 2010 and the experiment lasted until 2015. All coffee plants (*Coffea arabica* L.) were of the dwarf variety Caturra, with planting distances of 2 m between coffee rows and 1 m between plants within the row (5,000 plants ha^-1^). From 2010 to 2013, all coffee plants received the same management protocol, provided by the farm with three yearly applications of fertilizers, three of fungicides and other pesticides, and three of herbicides. From 2013 to 2015 only fungicide applications varied, depending on the treatments defined for this study. The experiment had six treatments, each one consisting in a particular sequence of chemical pesticide applications, including fungicides and insecticides (Fig 2). Insecticides were applied only in 2013. Given that the incidence of pests was low, insecticide applications were suspended the following years, which, in addition, allowed us to avoid the use of highly toxic products. Control of coffee berry borer (*Hypothenemus hampei* Ferrari) in the entire experimental parcel was achieved by using traps with chemical attractants. Each treatment had four randomized plots, representing a completely randomized design. Each plot had five rows and six plants per row, where external rows and plants were defined as borders. In the three central rows, we marked six plants and three branches per plant for measurements. One coffee plant pruning per year was performed in 2014 and 2015 to remove exhausted orthotropic stems, leaving two to four productive orthotropic stems per plant.

Treatments were designed for two specific objectives: (i) to generate different levels of injuries in different years in order to calculate yield losses through comparisons of treatments, i.e., by experimentation; and (ii) to generate variability of data among coffee plants regarding yield components, pest and disease injuries, and yields, to be used in the statistical modeling.

### Measurement and calculations of the variables studied from 2013 to 2015

We quantified several different yield components, yields, pest and disease injuries, and dead productive branches over all the years of the study, except for the latter, which were not measured in 2013 (Table 1). Basic statistical measures were calculated for these variables for each year.

Yield losses were expressed in units of the product or in percentages [1]:

$$Yloss=Yatt-Yact\;Yloss\;(\%)=\frac{Yatt-Yact}{Yatt}\times 100$$

where Yloss is yield loss in grams of fresh coffee cherries per plant (g plant^-1^), Yloss (%) is yield loss in percentage, Yatt is attainable yield, and Yact is actual yield.

**Table 1:** Variables characterized in the coffee experimental parcel, Turrialba, Costa Rica.

Variables	Descriptions	Time of assessments
**Yield components**		
Stems	Number of productive orthotropic stems per plant	All these variables were quantified before the beginning of the harvest season, when small fruits were already visible
Branches	Number of productive branches per plant	
Fruiting nodes	Number of fruiting nodes per plant	
Fruits per node	Number of fruits per node each 25 fruiting nodes; then averaged	
Coffee yield	Grams of ripe fresh coffee cherries per coffee plant	Harvests every 15 days
**Pests and diseases of leaves**		
sAUDPC of:	Incidences for each pest and disease per plant were calculated based on the accumulated number of infected/infested leaves with respect to the total of accumulated leaves at each assessment date	Infected/infested/healthy leaves were counted monthly
Coffee leaf rust	$AUDPC=\sum_{i=1}^{n-1}\frac{I_{i}+I_{i+1}}{2}\times (t_{i+1}-t_{i})$	
Brown eye spot	where *AUDPC*: area under the disease/pest progress curve; *I*_*i*_: incidence of a given pest or disease at the *i*^th^ measurement; *t*_*i*_: time (in days) of the *i*^th^ measurement; n: total number of measurements	
Anthracnose	sAUDPC=AUDPC/Nd	
Coffee leaf miner	where *sAUDPC*: standardized area under the disease/pest progress curve; *Nd*: total number of days of the assessment period [18]	
sAUDPC P&D	sAUDPC P&D: represents the sAUDPC of all pests and diseases together	
sAUPC of severity	A scale from 0 to 6 (the higher the number, the higher the severity), based on the size and number of symptoms on leaves, was used to assign a level of overall severity to marked branches. Averages of severities were calculated per plant, and then standardized areas under the progress curve (sAUPC) of severity were calculated.	Severity was assessed monthly for all visible injuries
Dead branches	Number of dead productive branches per plant	Quantified at the end of the coffee harvest period

Coffee leaf rust (*Hemileia vastarix* Berkeley and Broome); brown eye spot (*Cercospora coffeicola* Berk and Curtis); anthracnose (*Colletotrichum spp*.); coffee leaf miner (*Leucoptera coffeella* Guérin-Mèneville).

### Effects of treatments on coffee yields and calculation of yield losses through experimentation

Three-year sequences of chemical treatments (T_(n-2, n-1, n)_) starting from 2011, 2012, and 2013, constructed based on the scheme of treatments (Fig 2) and shown in Table 2, were used to estimate primary and secondary yield losses. Yield losses were obtained by comparing the yield obtained in the third year (*n* = 2013, 2014 or 2015) of the completely protected treatment (TTT), i.e., the attainable yield, with the yield obtained in the third year of other sequences of chemical treatments, i.e., different actual yields.

**Table 2:** Number of plots and plants considered in the analysis, according to different three-year sequences of chemical treatments and quantification of yield losses.

Three-year sequence of chemical treatments T_(n-2, n-1, n)_	Last year of the three year sequence of chemical treatments						Total plots	Total plants	Quantification of yield losses in the third year
	2013		2014		2015				
	plots	plants	plots	plants	plots	plants			
TTT^Yatt^	12	72	8	48	4	24	24	144^**†**^	
TTN	12	72	4	24	4	24	20	120^**†**^	TTT-TTN → 1ry Yloss^I^
TNT			4	24	4	24	8	48^**†**^	TTT-TNT → 2ry Yloss^II^
TNN			8	48			8	48^**†**^	TTT-TNN → 1ry + 2ry Yloss^III^
NTT					4	24	4	24	
NNT					4	24	4	24	
NNN					4	24	4	24	
Total	24	144	24	144	24	144	72	432	

*n*: year; T: treated with pesticides; N: no pest and disease control.

A treated plot (T) in a given year in the experimental parcel implies that reducing factors (RF) = 0 (Fig 1), because no pests and diseases or dead branches were expected; on the contrary, a no-treated plot (N) implies that reducing factors >0. For instance, the three-year sequence TNT implies that RF_(n-2)_ = 0, RF_(n-1)_ >0, RF_(n)_ = 0. The difference between the attainable yield (Yatt, obtained in TTT) and the actual yield in TNT in year *n* represents a secondary yield loss (Yloss) in year *n* caused by pest and disease injuries of year *n-1*.

^†^Number of plants used to test the effects of sequences of chemical treatments with the Model T.

All losses are quantified on year n;

^I^: primary losses resulting from the year *n* injuries;

^II^: secondary losses resulting from the year *n-1* injuries;

^III^: primary and secondary losses resulting from the years *n* and *n-1 injuries*.

We applied a linear mixed model for the analysis. The sequence of chemical treatments in the last three years (T_(n-2, n-1, n)_) was declared as the fixed effect, which included the sequences TTT, TTN, TNT, and TNN. Given that these sequences were present during several years (submitted to the biennial behavior of coffee production) and there were soil heterogeneity conditions among plots in the experimental parcel (especially the acidity of soil), the year and the plot were declared as random effects:

$$Yield\_Plant∼T_{\left(n-2,\;n-1,\;n\right)}+(1\;|\;Year)+(1\;|\;Plot)$$ (Model T)

where T_(n-2, n-1, n)_ is the statistical treatment (a three-year sequence of chemical treatments) and Yield_Plant is the coffee yield per plant in the third year.

The sequences NTT, NNT, and NNN were not included in the analysis because yields for the third year of these sequences were available only in the last year of the experiment (2015). The year 2015 was a year of low production in all of the experimental parcel (similar yields for all treatments). We checked that the inclusion of such sequences with the proposed Model T would have introduced a statistical unwanted bias in the results.

Model T was run in the program R [19] with the package ‘lme4’ [20], using the chi-squared test to determine significant effects of the three-year sequence of chemical treatments (*P*<0.05) on coffee yields of the third year. The normal of the data was verified by inspecting a histogram of the residuals. Significant differences between yields of the third year were analyzed using the LSD test, with the package ‘predictmeans’ [21, 22].

### Statistical modeling for the identification of the most important predictors of primary and secondary coffee yield losses

We used all variables showed in Table 1 as predictors of coffee yield. According to our conceptual model (Fig 1), the actual yield of a specific year is a direct function of yield components, pest and disease injuries, and dead productive branches for that year, and also the result of the indirect effect of the same predictors of the previous year. To take into account the chain of effects described in Fig 1, we applied piecewise structural equation modeling (PiecewiseSEM) [23]. PiecewiseSEM is a confirmatory path analysis that works with two or more linear models in order to test direct and indirect effects for estimation of the final response variable—in our case, yields. PiecewiseSEM permits testing whether the equations used to explain intermediate and final response variables are independent and a reflection of the true paths involved, for which *P*-value must be higher than the significance threshold (we chose α = 0.05) to ensure a good fit of the modeling. PiecewiseSEM also provides the Fisher’s C statistic and the likelihood degrees of freedom (K) to calculate the Akaike’s information criterion (AIC = C + 2K) [23].

Given that this modeling approach considers the influence of predictors of the previous year, it was necessary to use data of two consecutive years for each plant. We used data from 2014 and 2015 only, as no information was available for 2011 and 2012, and the number of dead branches was not assessed in 2013. Contrary to previous analysis, no treatments were excluded. However, since the estimation of yield losses makes sense only if plants have yield components >0, only plants with yield components >0 were used. Models were then constructed with data of only 82 plants out of the original 144.

We used linear mixed models to estimate the actual yield for 2015 and to model intermediate predictors influenced by those of the previous year (2014); in all cases, the plot was declared as random effect. The list of models included in PiecewiseSEM was as follows:

$${Yact}_{\left(n\right)}∼{Yield\;components}_{\left(n\right)}+{sAUDPC}_{\left(n\right)}+{DeadB}_{\left(n\right)}+(1\;|\;Plot)$$ (Main model)

$${Yield\;components}_{\left(n\right)}∼{Yield\;components}_{\left(n-1\right)}+{DeadB}_{\left(n-1\right)}+(1\;|\;Plot)$$

$${sAUDPC}_{\left(n\right)}∼{sAUDPC}_{\left(n-1\right)}+{Yield\;components}_{\left(n\right)}+(1\;|\;Plot)$$

$${DeadB}_{\left(n\right)}∼{sAUDPC}_{\left(n\right)}+{Yield\;components}_{\left(n\right)}+(1\;|\;Plot)$$

where Yact is actual coffee yield per plant; sAUDPC is the standardized area under the disease progress curve of pests and diseases (we included the sAUDPC of each pest and disease individually and also the sAUDPC P&D -all pests and diseases together-); DeadB is the number of dead productive branches; (n) represents the year of interest (2015 in this case), and (n-1) represents the previous year (2014).

We ran the PiecewiseSEM on this list in the software R [19] with the package ‘piecewiseSEM’ [23]. Different combinations of predictors in the models were tested, verifying that the *P*-value was *P* >0.05 to ensure a good fit, and suppressing predictors with no significant effects (*P* >0.05) or with very low effects (i.e., low coefficients <0.001) in each test. Which means that different lists of models were run until we selected the best list, based on two criteria: the list that showed the lowest values of AIC and the highest values of R^2^ provided by the PiecewiseSEM. Finally, based on the significance and on the coefficient of each predictor involved in the best list, also provided by the PiecewiseSEM procedure, we identified the most important predictors of yields, and of primary and secondary yield losses.

## Results

### Statistical description of the variables studied from 2013 to 2015

The most observable differences among variables across years happened in 2014 when the yield components, coffee yields, and dead productive branches were noticeable higher, especially in comparison with 2015, illustrating the biennial behavior of coffee production (Table 3). The most important diseases were coffee leaf rust and brown eye spot, according to sAUDPC values. Both diseases had wide ranges during the three years of measurements, especially in 2015 when some plants reached the 100% of sAUDPC. The other disease (anthracnose) and the insect coffee leaf miner had low levels (<7% on average). The overall severity of pest and disease attacks appeared to be important especially in 2014 and 2015, when the maximum values were between 5 and 6, which means that branches had most of the leaves with large symptoms caused by pests and diseases (scale 5) or were already dead (scale 6).

**Table 3:** Basic statistics of the variables studied in the coffee experimental parcel, Turrialba, Costa Rica.

Variable	2013		2014		2015	
	Mean±SD	Range	Mean±SD	Range	Mean±SD	Range
NS (number plant^-1^)	3±1	1–5	3±1	1–5	3±1	0–5
NPB (number plant^-1^)	145±77	64–433	97±42	0–209	25±32	0–134
NFN (number plant^-1^)	421±238	9–1158	500±329	0–1901	129±181	0–796
NF (number node^-1^)	4±1	2–7	3±1	0–6	1±1	0–4
Coffee yield (g plant^-1^)	2172±1354	5–7295	2416±1859	0–11600	680±1038	0–4862
sAUDPC_Rust (%)	28±14	3–71	36±14	0–71	34±15	0–100
sAUDPC_Cerc (%)	26±10	5–58	22±9	3–46	23±12	0–100
sAUDPC_Ant (%)	5±4	0–19	6±5	0–23	3±4	0–17
sAUDPC_Min (%)	1±1	0–8	4±4	0–15	2±3	0–12
sAUDPC_All (%)	59±10	32–88	67±9	44–96	58±12	24–100
sAUPC_Sev (scale)	2.7±0.6	1.4–4.4	3.6±0.7	2.1–5.5	2.9±0.8	1.5–5.8
DeadB (number plant^-1^)	46±26	14–114	26±31	0–193	8±6	0–29

SD: standard deviation; NS: number of productive orthotropic stems; NPB: number of productive branches; NFN: number of fruiting nodes; NF: number of fruits; Coffee yield: grams of fresh coffee cherries; sAUDPC: standardized area under the disease progress curve; Rust: coffee leaf rust; Cerc: brown eye spot; Ant: anthracnose; Min: coffee leaf miner; All: all pests and diseases; sAUPC_Sev: standardized area under the progress curve of severity; DeadB: number of dead productive branches

### Effects of three-year sequences of chemical treatments on yield losses

**Fig 3:**
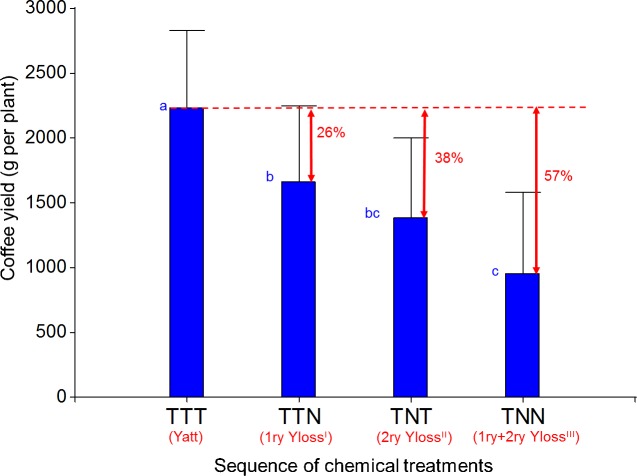
Yields and primary and secondary yield losses resulting from the sequences of chemical treatments TTT, TTN, TNT, TNN. T: treated with pesticides; N: no control of pests and diseases. Different lowercase letters between bars indicate significant differences (*P*<0.05) between coffee yields. Red arrows and numbers in percentages represent the yield losses. Yatt: attainable yield; Yloss: coffee yield loss; ^I^: primary losses resulting from the current-year injuries (year n); ^II^: secondary losses resulting from year n-1 injuries; ^III^: primary and secondary losses (total losses) resulting from years n and n-1 injuries.

The effects of the sequences TTT, TTN, TNT, and TNN (Model T) on coffee yield in the third year were significant (*P*-value = 0.0009) (Fig 3). The yield of the sequence TTT (the attainable yield), with 2235 g fresh coffee cherries plant^-1^, was the highest and significantly different from the other yields, which allowed us to quantify yield losses. The largest yield loss was the total yield loss (primary + secondary yield losses = 57%) as a consequence of no pest and disease control in the current and in the previous year (TNN). Primary yield losses (TTN) were high (26%), and secondary yield losses, due to previous-year injuries (TNT), resulted in even higher losses (38%).

### The most important predictors of primary and secondary coffee yield losses

**Fig 4:**
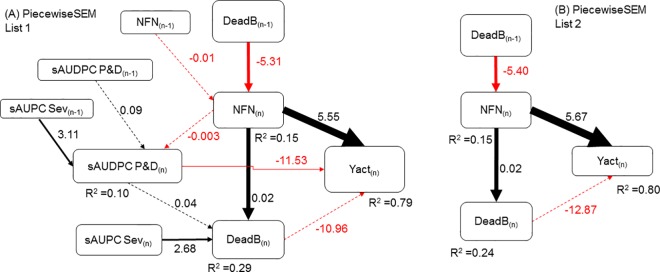
**Structural equation models for the estimation of actual coffee yield for Piecewise List 1 (A) and Piecewise List 2 (B) presented in Table 4**Yact: actual coffee yield per plant; NFN: number of fruiting nodes per plant; DeadB: number of dead productive branches per plant; sAUDPC P&D: standardized area under the disease progress curve of all pests and diseases together; sAUPC Sev: standardized area under the progress curve of severity; _(n)_: current year (2015); _(n-1)_: previous year (2014). Arrows represent relationships among variables; black arrows denote positive relationships and red arrows denote negative relationships; arrows for no significant paths (*P* >0.05) have dashed lines; arrows with significant paths (P <0.05) have continuous lines. The numbers near the arrows are the regression coefficients. The thickness of the significant paths was scaled based on the magnitude of the standardized regression coefficients (not shown in the figures).

We found two lists of linear mixed models that fitted well in the PiecewiseSEM (*P* >0.05). The first list (List 1) was the largest, with models for all the predictors that direct or indirectly affected actual yield, including yield components, pest and disease injuries, and severity and number of dead productive branches of current and previous years. The second list (List 2) was a simplified modeling, including only yield components and number of dead productive branches in the models (Table 4). The significance of the variables and the magnitude of their influences (coefficients), for both List 1 and List 2, are shown in Fig 4. From List 1, it can be deduced that effects within a specific year and across years explained in the conceptual model (Fig 1) are validated. This modeling however used several predictors (variables) which are not easily measurable in the field (e.g., sAUDPC of pests and diseases), and which were not significant in the modeling. Therefore, we used the List 2 to determine the most significant and easily measurable yield predictors. In addition, List 2 exhibited a lower AIC value than List 1 (Table 4).

**Table 4:** Models for the estimation of actual coffee yields in 2015 (g of fresh coffee cherries per plant) with data of 2014 and 2015, through Piecewise structural equation modeling

Number of list	Linear mixed models for the PiecewiseSEM	Indicators for the PiecewiseSEM			
		Fisher’s C	K	AIC	*P*-value
List 1	Yact_(n)_ ~ NFN_(n)_+DeadB_(n)_+sAUDPC P&D_(n)_+(1|Plot) **Main model**	36.03	23	82.03	0.21
	NFN_(n)_ ~ NFN_(n-1)_+DeadB_(n-1)_+(1|Plot)				
	sAUDPC_All_(n)_ ~ sAUDPC P&D_(n-1)_+sAUPC_Sev_(n-1)_+NFN_(n)_+(1|Plot)				
	DeadB_(n)_ ~ sAUDPC P&D_(n)_+sAUPC_Sev_(n)_+NFN_(n)_+(1|Plot)				
List 2	Yact_(n)_ ~ NFN_(n)_+DeadB_(n)_+(1|Plot) **Main model**	5.20	13	31.20	0.27
	NFN_(n)_ ~ DeadB_(n-1)_+(1|Plot)				
	DeadB_(n)_ ~ NFN_(n)_+(1|Plot)				

Yact: actual coffee yield per plant; NFN: number of fruiting nodes per plant; DeadB: number of dead productive branches per plant; sAUDPC P&D: standardized area under the disease progress curve of all pests and diseases; sAUPC_Sev: standardized area under the progress curve of severity; _(n)_: current year (2015); _(n-1)_: previous year (2014); K: likelihood degrees of freedom; AIC: Akaike’s information criterion.

Thereby, the most important and useful predictors of yield, considering influences of at least two years, were: the number of fruiting nodes of the current year characterizing yield components, and the number of dead productive branches of both the current year and the previous year, characterizing reducing factors of yield (List 2 in Fig 4). Dead productive branches of the previous year were considered as predictors of secondary yield losses since they had negative effects on the number of branches bearing fruiting nodes of the following year, representing a one-year delayed indirect effect on the actual yield. Dead productive branches of the current year were considered as predictors of primary yield losses since they had direct negative effects on the number of fruiting nodes remaining at the harvest time, i.e. the actual yield.

## Discussion

### Primary and secondary yield losses

Our study shows that both primary (26%) and secondary yield losses (38%) caused by foliar pests and diseases can be severe in a perennial crop. Efforts to estimate yield losses have increased in the last decades, but most of them concentrated in annual crops and focused on primary yield losses. On annual crops, yield losses due to pests and diseases were estimated from 24% to 41% in rice in Asia [24], 5% to 96% in potatoes in France [25], and up to 7 t ha^-1^ in wheat in France [26]. In perennial crops, studies on apple and other stone fruits reported yield losses that reached up to 5% in the Netherlands [27]. In Thailand yield losses of cotton were estimated up to 100% [28]. In coffee, yield losses were reported from 13% to 45% in Brazil [29]. At a global scale, yield losses ranging from 20% to 40% were quantified in rice, wheat, barley, maize, potatoes, soybeans, cotton, and coffee in different countries and regions [3, 11]. In all these cases, yield losses were assessed in the current year, and therefore we assumed that they were primary yield losses. The ranges of these primary yield losses are wide, depending on the type of crop/variety and pests and diseases. In our study, we provided new quantitative data indicating that primary yield losses on a perennial crop such as coffee can reach 26% due to a set of foliar pests and diseases.

To our knowledge, our study was the first attempt to quantify secondary yield losses, which could have important implications for perennial crops if they are as high as the one we estimated for coffee (38%). In accordance with the hypothesis proposed by Avelino and colleagues [2, 30–32], our study confirms that the attacks of foliar pests and diseases have delayed impacts in coffee. We show that secondary yield losses are an important issue in perennial crops whose production depends on the growth of organs in the previous years. In annual crops, secondary yield losses are principally related to the inoculum of pathogens in the soil or to infected/infested seeds [10]. However, annual-crop farmers normally select or buy the best and healthiest seeds, do rotations, or sometimes disinfect soil before sowings; therefore, expected secondary losses should be quite low. On the opposite, in perennial crops, secondary yield losses, resulting from previous year damages, cannot be avoided. Losses over several consecutive years are even expected, which can only be reduced by implementing appropriate practices to recover plant growth.

The finding that the number of dead productive branches of a previous year is the most important reducer of the number of fruiting nodes of the next year (Fig 4) has implications for the management of coffee plant architecture. In areas under disease pressure, it would be better to promote more developing branches (for the next year) and more productive branches without many fruiting nodes per branch than to have few productive branches and many fruiting nodes per branch. That way the risk of losses due to the death of branches would be reduced.

**Fig 5:**
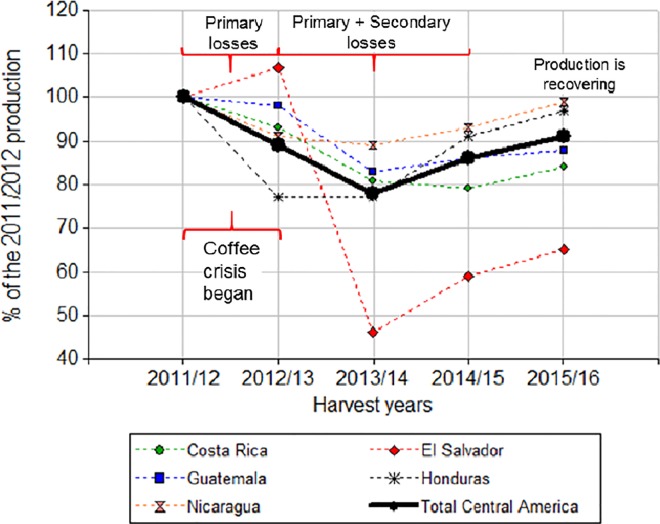
Evolution of coffee production in Central America as a percentage of the production in the harvest year 2011/2012. Curves were constructed with data of the International Coffee Organization [33]; the production in 2011/2012 (before coffee crisis) was taken as the reference, corresponding to 100%.

Our findings can also contribute to the explanation of severe reductions in productivity in perennial crops, as happened during the coffee crisis in Central America. This crisis started in 2012 as a combination of the outbreak of coffee leaf rust and inefficient management of coffee plantations [2]. From that year, the production decreased in two successive years in most of the countries. By analyzing the evolution of coffee production from the year prior to the crisis to the present, we deduced that: the total production in Central America in the harvest year 2012–13 decreased about 10% with respect to 2011–12, which could be attributed in part to the primary losses as a consequence of the first impacts of the disease; but the most severe impacts (defoliation and/or death of branches or plants) were seen in the harvest year 2013–14, with a 20% of reduction with respect to 2011–12, reflecting the seriousness of secondary losses (Fig 5). The production started to recover in the harvests 2014–15 and 2015–16, in part due to control actions conducted at national and regional levels and to pruning operations as stumping (total cut of coffee plants at 30–40 cm from the ground level) conducted by farmers in 2013, with production recovering two or three years later [2, 16].

### Methodological aspects of experimentation and modeling

Our experimental approach was useful in overcoming the difficulties of quantifying yield losses. Most attempts to assess yield losses were based on statistical relationships between yields and descriptors of specific pests or diseases in empirical models. However, these relationships can be masked or weakened by several confounding factors such as interactions among pests and diseases, interactions among these pests and diseases and other factors impacting on production, such as physiological effects of high temperatures, soil acidity and overbearing disease (dieback of plants) [4, 34]. For instance, in the case of coffee leaf rust, researchers assumed that the disease did not cause primary yield losses because its incidence was positively related to fruit load and even to the yield of the current year (the higher the yield, the higher the coffee rust incidence) [31]. Our experiment, based on treatment sequences, enabled us to estimate both primary and secondary yield losses.

In addition, our modeling approach was useful to identify the number of dead productive branches as a predictor of the impact of pests and diseases on yield and at the same time as a pathway (driver) that leads to yield losses. Models to estimate yield losses should be improved by adding this kind of variables that represent mechanistic links [4, 8, 35]. The modeling also revealed that pests and diseases were not important individually for the estimation of coffee yield. Only sAUDPC of all pests and diseases together and their overall severity fitted well in the PiecewiseSEM, confirming the relevance of the assessment of injury profiles, defined as a given combination of injury levels caused by a set of pests and diseases in a crop cycle [8].

For coffee, there are few but important physiological process-based (mechanistic) models that could benefit from our findings. One of these models is capable of simulating plant growth and production under agroforestry systems in Central America. Another model was developed in Brazil and predicts growth of vegetative parts and yield components, and incorporates dynamics of the coffee berry borer and its natural enemies [36]. These mechanistic models have a general value and can be used in different conditions, as was done in Colombia using the last model [37]. Our findings on the importance of dead productive branches as yield-reducing factors can possibly be incorporated into such models to assess yield losses due to pests and diseases.

### Practical applications considering management and other production factors

Similar approaches of experimental design and modeling used in this study, could be applied to other perennial crops to assess primary and secondary yield losses. The data of yield losses can be used to quantify economic losses in different agroecosystems, as well as to assess the effectiveness of pest and disease regulation as an ecosystem service, in terms of reduced or avoided losses [6, 7, 12].

Furthermore, once yield losses are quantified, then the main causes can be analyzed, shedding light on how to improve production systems. Several studies, for instance in wheat [26, 38], rice [39], and coffee [7], highlight the importance of identifying production factors (environment, topography, soil, associated plant biodiversity) that influence injury profiles, but few studies related production factors directly to yield losses, with very limited exceptions—such as a yield loss assessment due to pests and diseases in rice [24]. In the case of coffee and other crops that can be grown in agroforestry systems, the associated plant biodiversity and shade cover are probably important production factors that need to be considered in crop-loss assessments due to their influence on crop growth and fruit load, pest and disease injury levels [34], and branch dieback [17].

There are also several studies on the reduction of crop yields, referred as “yield gap”. The yield gap, in a broad definition, is “the difference between two levels of yield,” which can be chosen according to particular objectives [40]. Some studies quantified the yield gap as the difference between the site-specific potential yield (obtained with no limitations of nutrients and water, or pest or disease attacks) and the observed actual yield [41–44]. Others quantified the yield gap as the difference between the maximum attainable yield identified in a whole region and the actual yield on a given farm [45–47]. In both cases, yield gaps can be considered as yield reductions caused not only by pests and diseases but also by other production factors. Like yield losses, yield gaps were estimated mainly for annual crops, and few for perennial ones. One study quantified coffee-yield gaps from 45% to 57% in Uganda, identifying poor management practices, poor soil fertility, and the coffee twig borer as the main causes [46]. From this study, we can hypothesize that coffee yield losses also vary according to different production factors and management.

The most important and useful predictors identified in this study could be considered as indicators of yield losses, and used to estimate yield losses in farms of different coffee-growing areas. Measurements can be easily done at three critical times: dead productive branches just after the current harvest, fruiting nodes after the next flowering, and finally, the dead productive branches at the end of the next harvest. The data needed to estimate primary and secondary yield losses can therefore be obtained in the lapse of one year. These predictors could be included in linear mixed models to estimate actual yields, using random effects to consider the influence of local production factors. The random effects can improve the estimations, especially when working in large areas (with diverse production factors). Once the equations are obtained, the predictors representing reduction factors (dead productive branches in this case) can be set as “zero” to estimate attainable yields, and then calculate yield losses (attainable yield–actual yield). Such attainable yields are the yields without negative impacts of the current and the previous year, making possible to calculate both primary and secondary yield losses, as suggested in the conceptual model (Fig 1).

Once the models and equations with their respective coefficients are defined for a given coffee-growing area, they can be used to estimate the yield losses that have already occurred as well as to predict what to expect in the next harvest. Such predictions, along with the identification of the main causes of yield losses, can act as motivators for improvement of the production system, because the user (farmer, technician, or researcher) will know how much losses could be avoided and how much could be gained by implementing the necessary measures in the system [1].

## Conclusions

Our work demonstrates that foliar pests and diseases in a perennial crop can lead to high primary yield losses and even higher secondary yield losses. To our knowledge, this research is the first to quantify secondary yield losses in a perennial crop, revealing the importance of its assessment since high secondary yield losses, as in the case of coffee, can have severe consequences for crop production over several years.

Fruiting nodes and dead productive branches were the most important and useful predictors of coffee yields and yield losses. Both predictors had significant effects in the modeling and are indicators easy to measure in the field.

Our research contributes to the field of crop losses, providing experimental and modeling approaches that could be used in perennial crops to estimate primary and secondary yield losses. The usefulness of this contribution for further studies is that once the yield losses are estimated, economic losses can be deduced and the main causes of losses identified, allowing the farmer to take corrective actions.

## Supporting Information

S1 File Minimal data set of the study. (XLSX)
